# Supplementary material for: An Upgrade on the Surveillance System of SARS-CoV-2: Deployment of New Methods for Genetic Inspection
Source: Int J Mol Sci. 2022 Mar 15;23(6):3143. doi: 10.3390/ijms23063143 (PMC8950365; doi:10.3390/ijms23063143)
Supplement: Supplementary file 1 [file ijms-23-03143-s001.zip › ijms-1545983-supplementary.pdf]

Supplemental Materials:

An Upgrade on the Surveillance System of SARS-Cov-2: Deployment of New Methods for Genetic Inspection

Table S1: Master Mut Kit interpretation

Reaction 1				Reaction 2				Mutation pattern	Variant
K417N	N501Y	E484K	R346K	L452R/Q	Del 69/70	D253N	T478K		
FAM	HEX	CAL Fluor Red 610	Quasar670	FAM	HEX	CAL Fluor Red 610	Quasar670		
-	+	-	-	-	+	-	-	N501Y & Del 69/70 mutations detected	Alpha
+	+	+	-	-	-	-	-	K417N, N501Y & E484K mutations detected.	Beta
V	+	+	-	-	-	-	-	N501Y & E484K mutations detected. Some virus can contain K417N	Gamma
-	-	-	-	+	-	-	+	L452R & T478K mutations detected	Delta
-	-	-	-	+	-	-	-	L452R mutation detected	Epsilon/Kappa
-	-	+	-	-	+	-	-	E484K & Del 69/70 mutations detected.	Eta
-	-	+	-	-	-	-	-	E484K mutation detected.	Iota
-	-	-	-	+	-	+	-	L452Q & D253N mutations detected.	Lambda
-	+	+	+	-	-	-	-	N501Y, E484K & R346K mutations detected	Mu
+	+	-	V	-	+	-	+	Del 69/70, K417N, T478K & N501Y mutations detected. Some virus can contain R346K.	Omicron BA.1
+	+	-	-	-	-	-	+	K417N, T478K & N501Y mutations detected.	Omicron BA.2
-	-	+	-	-	-	-	-	E484K mutation detected.	P.2
-	-	-	-	-	-	-	+	T478K mutation detected.	B.1.1.519

+: Mutation present, -: Mutation not present, V: Mutation present in some samples.

* Table has been modified from original to show only VOC, VOI and P.2 and B.1.1.519.

Master Mut Kit amplification plots.

The assay was performed in a CFX96 Real-Time System, from Bio-Rad. A single image is presented by each reaction. Images on the left correspond to Master Mut Kit Reaction 1 and images on the right correspond to Master Mut Kit Reaction 2. For sample M84, an extra image with both reactions is shown. Green plot correspond to FAM channel, blue to HEX channel, red plot represents CAL Fluor Red 610 channel and purple belongs to Quasar 670 (Q670) channel.

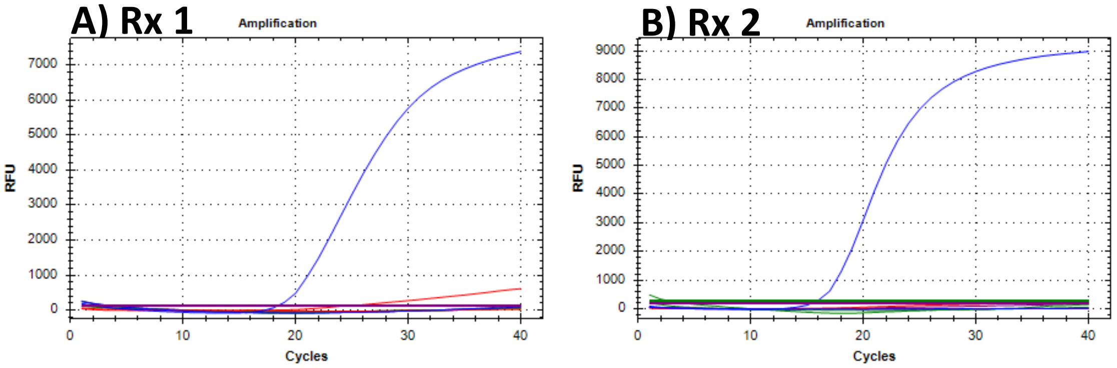

Figure S1: Master Mut Kit result for Alpha variant. A) Reaction 1. This sample contains N501Y (HEX channel). B) Reaction 2. Amplification plots of Del69-70 (HEX). Although it has been reported to contain E484K or L452R in addition to these two mutations, we have not found this pattern.

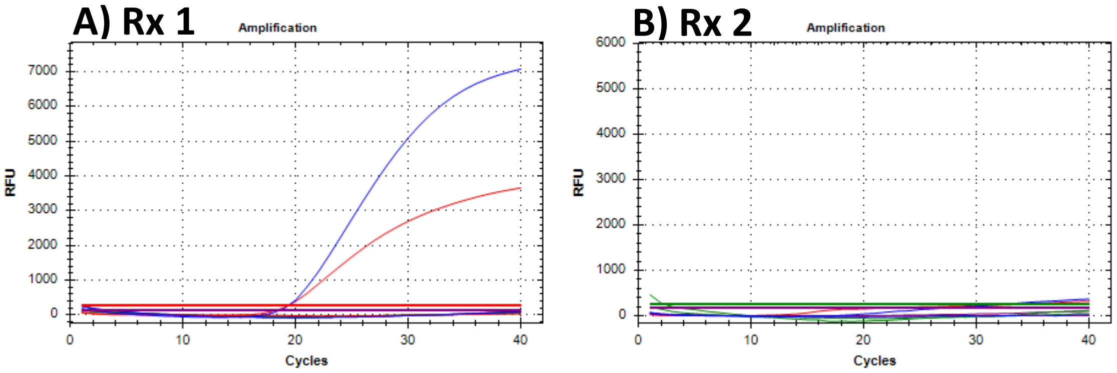

Figure S2: Master Mut Kit result for Gamma variant. A) Reaction 1. This sample contains N501Y (HEX channel) and E484K (CAL Fluor Red 610). B) Reaction 2. No amplification was detected.

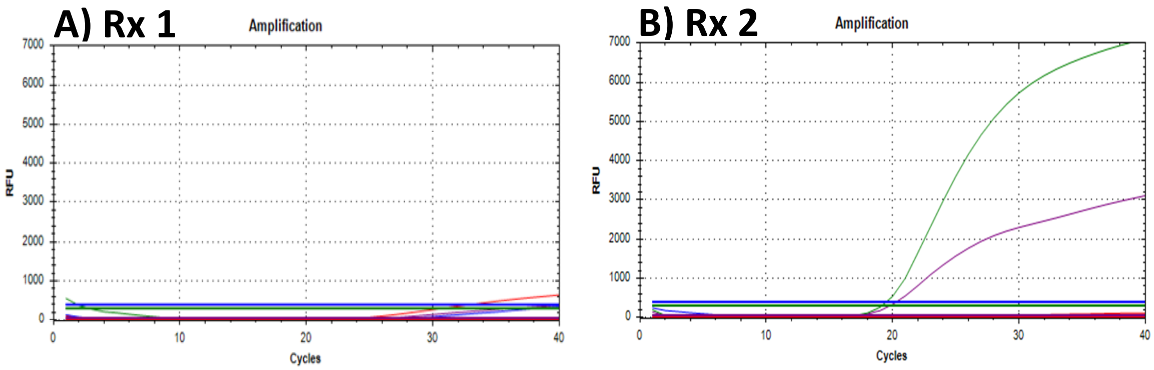

Figure S3: Master Mut Kit result for Delta. A) Reaction 1. No amplification detected. B) Reaction 2. Amplification plots of L452R/Q (FAM) and T478K (Q670).

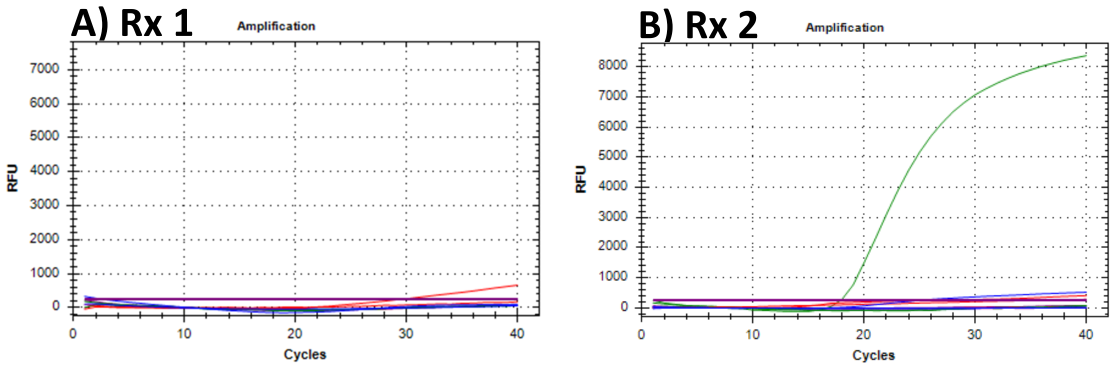

Figure S4: Master Mut Kit result for Epsilon/Kappa. A) Reaction 1. No amplification detected. B) Reaction 2. Amplification plot of L452R/Q (FAM).

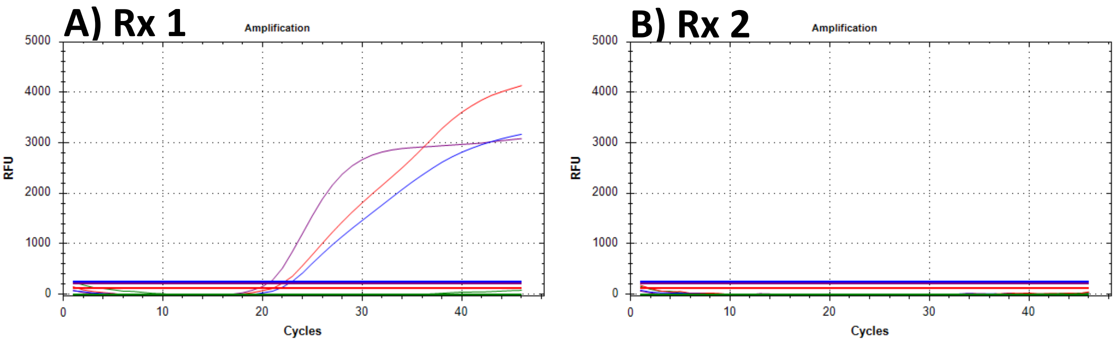

Figure S5: Master Mut Kit result for Mu. A) Reaction 1. This sample contains N501Y (HEX), E484K (CAL Fluor Red 610) and R346K (Q670). B) Reaction 2. No amplification detected.

.

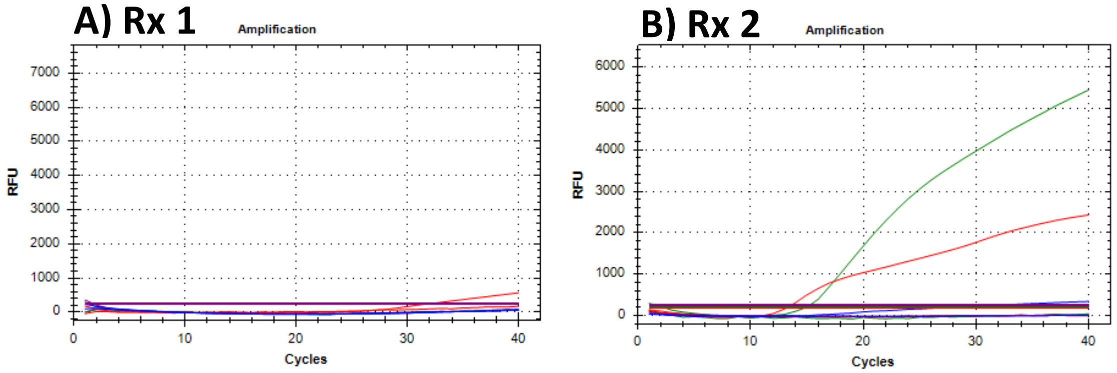

Figure S6: Master Mut Kit result for Lambda. A) Reaction 1. No amplification detected. B) Reaction 2. Amplification plots of L452R/Q (FAM) and D253N (CAL Fluor Red 610).

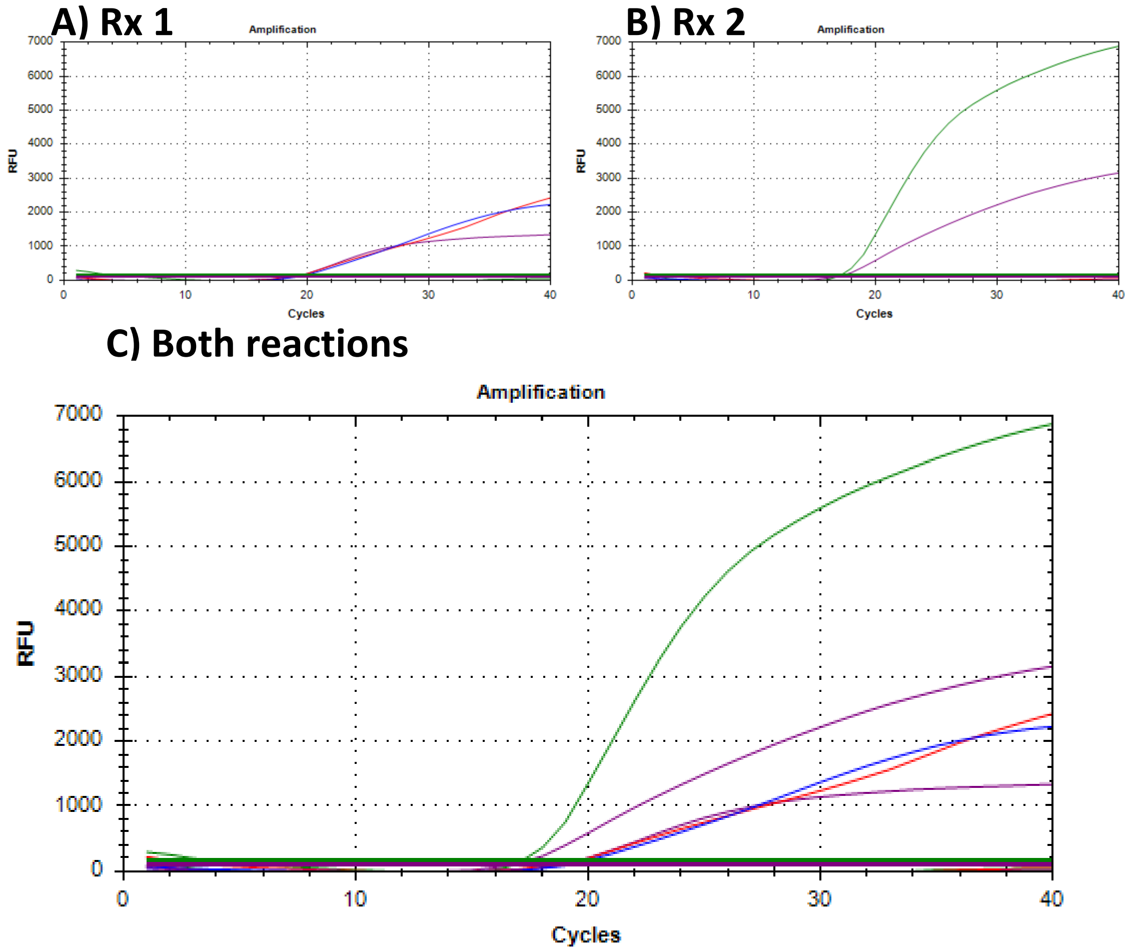

Figure S7: Master Mut Kit result for Sample M84. A) Reaction 1. This sample contains N501Y (HEX channel), E484K (CAL Fluor Red 610), and R346K (Q670 channel) B) Reaction 2. Amplification plots of L452R/Q (FAM) and T478K (Q670). C) Both reaction of M84 sample. The Cq for each mutation is: L452R/Q (Cq = 16.54), T478K (Cq = 16.57), N501Y (Cq = 19.1), E484K (Cq = 18.75) and R346K (Cq = 18.71).

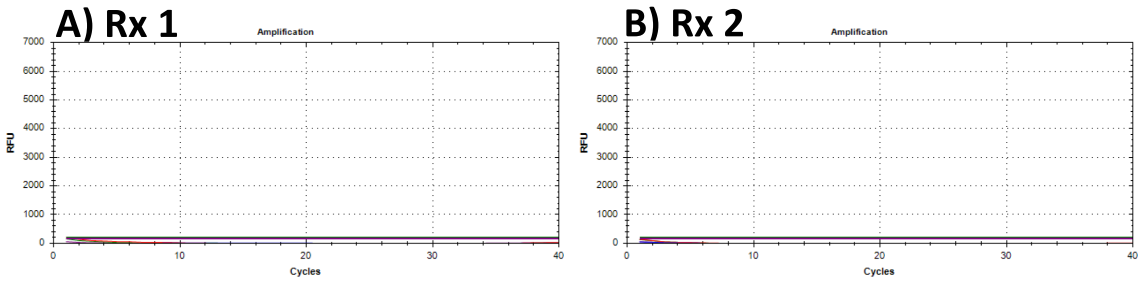

Figure S8: Results from a sample with no detectable mutations. The sequence of this sample (M40) was obtained through NGS and detected two mutations at the Spike gene (S477N and D614G). The nucleotide mutations were G22992A and A23403G, which didn´t interfere with the detection.

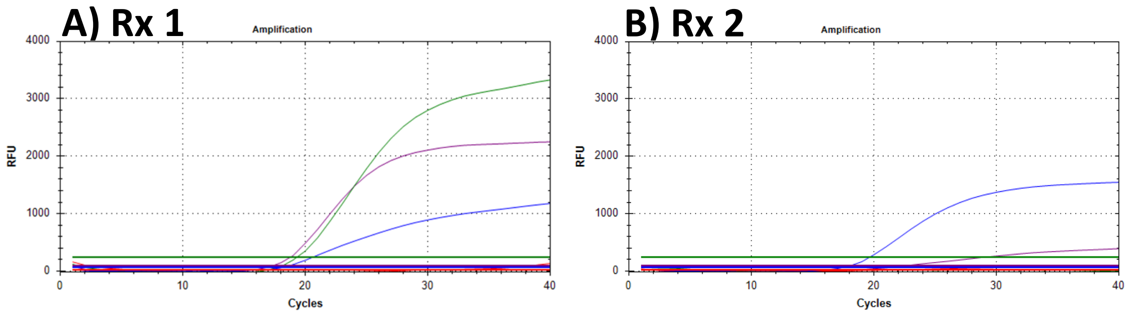

Figure S9: Master Mut Kit result for Omicron. A) Reaction 1. This sample contains K417N (FAM) N501Y (HEX) and R346K (Q670) B) Reaction 2. Amplification plots of Del69-70 (HEX) and a faint amplification in T478K (Q670).
